# Shortcomings of short hairpin RNA-based transgenic RNA interference in mouse oocytes

**DOI:** 10.1186/1477-5751-9-8

**Published:** 2010-10-12

**Authors:** Lenka Sarnova, Radek Malik, Radislav Sedlacek, Petr Svoboda

**Affiliations:** 1 Department of Epigenetic Regulations, Institute of Molecular Genetics of the AS CR, Videnska 1083, CZ-14220 Prague 4, Czech Republic; 2 Department of Transgenic Models of Diseases, Institute of Molecular Genetics of the AS CR, Videnska 1083, CZ-14220 Prague 4, Czech Republic

## Abstract

**Background:**

RNA interference (RNAi) is a powerful approach to study a gene function. Transgenic RNAi is an adaptation of this approach where suppression of a specific gene is achieved by expression of an RNA hairpin from a transgene. In somatic cells, where a long double-stranded RNA (dsRNA) longer than 30 base-pairs can induce a sequence-independent interferon response, short hairpin RNA (shRNA) expression is used to induce RNAi. In contrast, transgenic RNAi in the oocyte routinely employs a long RNA hairpin. Transgenic RNAi based on long hairpin RNA, although robust and successful, is restricted to a few cell types, where long double-stranded RNA does not induce sequence-independent responses. Transgenic RNAi in mouse oocytes based on a shRNA offers several potential advantages, including simple cloning of the transgenic vector and an ability to use the same targeting construct in any cell type.

**Results:**

Here we report our experience with shRNA-based transgenic RNAi in mouse oocytes. Despite optimal starting conditions for this experiment, we experienced several setbacks, which outweigh potential benefits of the shRNA system. First, obtaining an efficient shRNA is potentially a time-consuming and expensive task. Second, we observed that our transgene, which was based on a common commercial vector, was readily silenced in transgenic animals.

**Conclusions:**

We conclude that, the long RNA hairpin-based RNAi is more reliable and cost-effective and we recommend it as a method-of-choice when a gene is studied selectively in the oocyte.

## Background

RNA interference (RNAi) is a sequence-specific mRNA degradation induced by double stranded RNA (dsRNA). Briefly, long dsRNA is processed in the cytoplasm by RNase III Dicer into 20 - 22 bp long short interfering RNAs (siRNAs), which are loaded on the effector RNA-induced silencing complex (RISC). siRNAs serve as guides for cleavage of complementary RNAs, which are cleaved in the middle of the duplex formed between a siRNA and its cognate RNA (reviewed in detail in [1]).

RNAi is a widely used approach for inhibiting gene function in many eukaryotic model systems. Compared to other strategies for blocking gene functions, RNAi provides several advantages. It can be used to silence any gene, it is fast, relatively simple to use, and its cost is reasonably low. RNAi is usually induced either by delivering siRNAs or long dsRNAs into cells or by expressing RNA-inducing molecules from a vector. A number of strategies was developed for tissue-specific and inducible RNAi, thus offering an attractive alternative to traditional gene targeting by homologous recombination.

**Figure 1:**
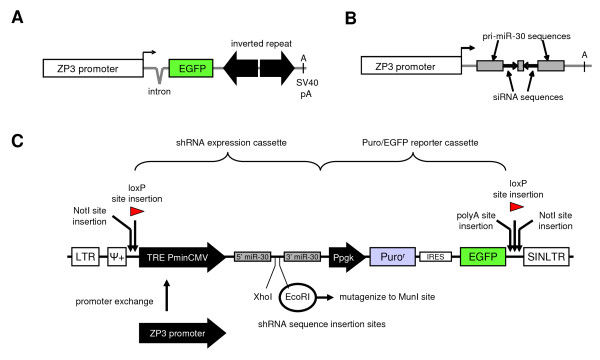
**Schematic representation of RNAi vectors**. **(A) **A typical RNAi transgene expressing long dsRNA hairpin under the control of oocyte-specific ZP3 promoter [5]. **(B) **A shRNA expressing cassette based on the endogenous human miR-30 precursor. **(C) **Highlighted features and adaptations of the pTMP plasmid to produce the expression cassette of the pZMP plasmid for transgenic RNAi in the oocyte.

RNAi became a favorable tool to block gene function also in mammalian oocytes. In fact, mouse oocytes were the first mammalian cell type where RNAi was used [2,3]. RNAi induced by microinjection of long dsRNA or siRNA into fully-grown germinal vesicle-intact (GV) oocytes is an excellent tool to study the role of dormant maternal mRNAs. These mRNAs are not translated before resumption of meiosis, so the stability of the protein product is not a factor influencing the efficiency of RNAi. In addition, resumption of meiosis can be delayed by compounds preventing reduction of cAMP levels in the GV oocyte, such as isobutylmethylxantine (IBMX) or milrinone, hence the period of mRNA degradation in microinjected oocytes can be prolonged for up to 48 hours [4]. The ability to target also genes translated during oocyte growth has been greatly enhanced by development of transgenic RNAi based on oocyte-specific expression of long dsRNA hairpin (Figure 1A, [5]). In comparison to the traditional conditional knock-out, transgenic RNAi is simpler, cheaper, and can produce phenotypes of different severity, depending on the knockdown level [5,6]. At least ten genes were efficiently suppressed in the mouse oocyte using a long hairpin-expressing transgene ([7] and P.S., unpublished results). Transgenic RNAi based on long RNA hairpin expression, however, has two limitations. First, cloning an inverted repeat needed for long RNA hairpin expression may sometimes be a difficult task. Second, long dsRNA efficiently induces a specific RNAi effect only in a limited number of cell types (reviewed in [7]). Endogenous RNAi manifested by the presence of endogenous siRNAs derived from long dsRNA, was found only in oocytes and embryonic stem (ES) cells, an artificial cell type closely related to cells of the blastocyst stage [8-10]. Because dsRNA longer than 30 bp has been reported to trigger the interferon response [11] and sequence-independent effects were observed in differentiated ES cells [12], induction of RNAi with expressed long hairpin RNA never acquired wider attention besides mouse oocytes.

We decided to develop and test a new transgenic RNAi vector for oocyte-specific short hairpin RNA (shRNA) expression, which would be compatible with RNAi vectors used in somatic cells and would be more versatile than the traditional transgenic RNAi design (Figure 1). First, a simple promoter swap would allow for using the same RNAi system for blocking genes in cultured cells or in tissues. Second, cloning shRNA-producing vector is easier when compared to cloning large inverted repeats. Third, a new vector would be compatible with different strategies to generate transgenic RNAi animals.

## Results

### Vector design

The RNAi targeting vector, named pZMP (Figure 1C) was based on pTMP and pLMP plasmids (Open Biosystems), which were selected as suitable starting vectors for producing a vector for transgenic RNAi in mouse oocyte. Vectors pTMP and pLMP allow for stable integration into the genome upon viral transduction and they carry suitable restriction sites for additional modifications. Furthermore, we needed a vector where shRNA expression would be driven by RNA polymerase II (pol II). The first shRNA systems were driven by pol III (reviewed in [13]). Pol II systems appeared later [14-17] since their development required better understanding of microRNA (miRNA) biology. miRNAs are genome-encoded small RNAs, which are loaded on the same effector complexes as siRNAs in mammalian cells [18].

Requirement for oocyte-specific expression dictated using a pol II-driven shRNA mimicking endogenous miRNA. The oocyte-specific expression of shRNA (Figure 1A) is controlled by the ZP3 promoter (hence pZMP), which is highly active during oocyte growth [19]. The transgenic cassette is flanked by LoxP sequences and NotI sites allowing for Cre-mediated insertion in the genome and simple release of the transgene from the plasmid for microinjection, respectively. Finally, the EcoRI site used for insertion of shRNA was mutated to MunI because there is another EcoRI site present in the ZP3 promoter. Since, MunI and EcoRI produce compatible overhangs the same oligonucleotides can be used for inserting shRNA into pTMP, pLMP and pZMP plasmids.

### Vector cloning and testing

**Figure 2:**
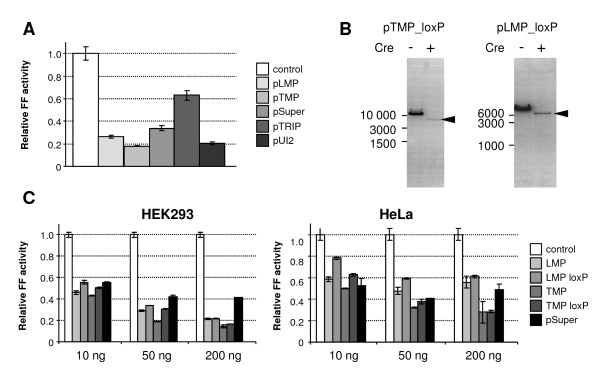
**Functional characterization of shRNA-expressing plasmids**. **(A) **HeLa cells were co-transfected with 50 ng of plasmids expressing shRNA targeting firefly luciferase, 200 ng of target pGL2 plasmid and 1 ng of phRL-SV40. Firefly luciferase (FF) activity normalized according to non-targeted *Renilla *luciferase activity is shown. Firefly luciferase activity in control sample (without a shRNA-expressing plasmid) was set to 1. Values are expressed as mean +/- SEM from samples transfected at least in triplicates. **(B) **pTMP and pLMP plasmids carrying loxP sites were transformed either to regular or Cre recombinase-expressing *E. coli *strains. Electrophoresis of isolated plasmid DNA is shown. The recombined plasmid after Cre-mediated recombination is marked by an arrow. **(C) **HeLa and HEK293 cells were co-transfected with 10-200 ng of plasmids expressing shRNA targeting firefly luciferase, 200 ng of target pGL2 plasmid, and 1 ng of phRL-SV40. Relative firefly luciferase activity compared to control cells is shown. Firefly luciferase activity in the control sample (omitting shRNA-expressing plasmid) was set to 1. Values are expressed as mean +/- SEM from samples transfected at least in triplicates.

First, we compared pTMP and pLMP vectors with three other shRNA vectors, to assure that both parental vectors would offer robust silencing. pTMP and pLMP essentially differ in the promoter controlling shRNA expression. pLMP uses the constitutively active 5'LTR promoter, while the pTMP vector uses a modified CMV promoter allowing for tetracycline-inducible expression. Using a published shRNA sequence targeting firefly luciferase [20], we generated five different vectors targeting firefly luciferase sequence, and compared their efficiency in transiently transfected cell lines (Figure 2A). Our results showed that pTMP and pLMP vectors induce RNAi efficiently, when compared to other shRNA vectors.

Next, we modified pLMP and pTMP plasmids by inserting linkers with LoxP and NotI sites, which flank the expression cassette (Figure 1C). The functionality of LoxP sites was tested in *E. coli *strain expressing Cre recombinase (Figure 2B) and we also verified that LoxP insertion has no effect on the efficiency of RNAi induced by these vectors (Figure 2C). Subsequently, the ZP3 promoter from the published transgenic RNAi cassette [5] was inserted in the pLMP vector (Figure 1C) and the NotI-flanked vector backbone was exchanged with the pTMP because it is modified to render the retrovirus-integrated 5' LTR transcriptionally inactive, in order to prevent interfering with the pol II promoter driving shRNA expression. The vector sequence was verified by sequencing. The functionality of PGK-driven puromycin-IRES-EGFP reporter was tested in cell culture.

### Mos shRNA selection

*Mos *dormant maternal mRNA was selected as the target for the new RNAi vector. Targeting *Mos *gene offers several advantages. First, *Mos *knock-out phenotype is manifested as sterility or subfertility, which is caused by parthenogenetic activation of eggs in otherwise normal animals [21,22]. This allows for simple scoring for the null phenotype and identification of potential non-specific effects of the PGK-driven reporter system in somatic cells. Second, maternal *Mos *has been targeted by microinjection of long dsRNA [2,3,23], siRNA [23] and by transgenic RNAi with long dsRNA [5,24], so there is a considerable volume of data for evaluating pZMP vector efficiency.

**Figure 3:**
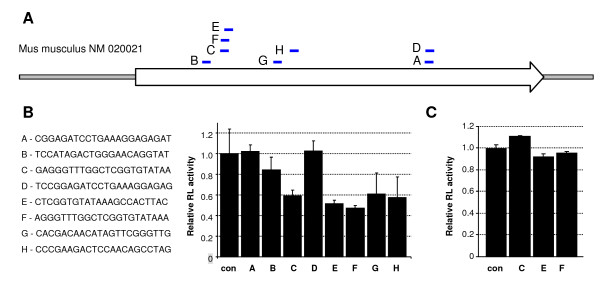
**Functional characterization of Mos-targeted shRNAs**.**(A) **A schematic position of *Mos*-targeting shRNAs within the *Mos *mRNA. The *Mos *coding region is represented by an arrow. **(B) **HeLa cells were co-transfected with 50 ng of pLMP_LoxP plasmid expressing various *Mos*-targeting shRNAs and 50 ng of target *Renilla *luciferase plasmid carrying a fragment of *Mos *gene in sense orientation in the 3' UTR, and 50 ng of pGL4-SV40. Relative *Renilla *luciferase (RL) activity normalized to co-transfected untargeted firefly luciferase is shown. RL activity in the control sample (no shRNA-expressing plasmid) was set to 1. Values are expressed as mean +/- SEM from samples transfected at least in triplicates. Mos_F shRNA cloned into pSUPER plasmid is shown for comparison. **(C) **Same experimental design as in (B) except *Renilla *luciferase with antisense *Mos *target sequence in 3'-UTR was used as a reporter.

To silence *Mos*, we designed eight different shRNA sequences located within the *Mos *coding sequence (Figure 3A). *Mos*-targeting siRNAs were predicted by RNAi Codex database [25], BIOPREDsi [26], RNAxs [27] and RNAi Oligo Retriever [28] tools. Best scored siRNAs predicted by different algorithms were inserted in pLMP and pTMP vectors in the form of shRNA and were subsequently experimentally tested to find the most efficient constructs.

A *Mos *fragment, carrying homologous sequences to selected shRNAs, was inserted in the 3'UTR of *Renilla *luciferase and resulting reporter was used to estimate the inhibitory potential of individual shRNAs (Figure 3B). We also tested the strand selection of most efficient shRNAs to verify that the desired shRNA strand is efficiently loaded on the RISC. In this case, we used a *Renilla *luciferase reporter with the cognate *Mos *target sequence inserted in the antisense orientation. Our results suggested that the *Mos *mRNA targeting siRNA strand is specifically loaded on the RISC complex, while the other strand (so-called "passenger strand") had a negligible effect on the reporter (Figure 3C). This indicated an efficient loading of the correct siRNA strand. Based on these data, we have chosen the Mos_F shRNA sequence for further experiments and inserted it into the pZMP plasmid. Then, NotI-flanked transgenic cassette was released and, after purification, the linearized DNA fragment was used for transgenesis by pronuclear microinjection into once-cell embryos.

### Analysis of transgenic mice

Upon embryo transfer, 56 founder (F_0_) mice were born. Six of these mice were positive for the transgene by PCR genotyping. One of the founder animals (#840) never transmitted the transgene into the F_1 _generation and one founder male (#900) did not produce any progeny. F_0 _mice from the remaining transgenic lines (#819, #835, #892, and #896) were fertile and transmitted the transgene. These lines were expanded and further examined. Interestingly, we noticed that the transgene transmission into the male progeny was reduced in all four lines (Table 1). Whether this unique sex-specific effect is caused by a particular transgene sequence, or is specific to disturbance of Mos expression [29,30], or is an effect of a hemizygous locus in a homozygous genetic background is unknown and is currently under investigation.

**Table 1:** Overview of F_1 _and F_2 _progeny of transgenic founder animals

Sex	Transgene	Number of pups in individual lines				Sum	%
				
		#819	#835	#892	#896		
**M**	**+**	**8**	**5**	**8**	**13**	**34**	**33.3%**
	
	-	19	12	16	21	68	66.6%

**F**	**+**	**20**	**4**	**20**	**15**	**59**	**54.6%**
	
	-	10	6	15	18	49	45.4%

**Figure 4:**
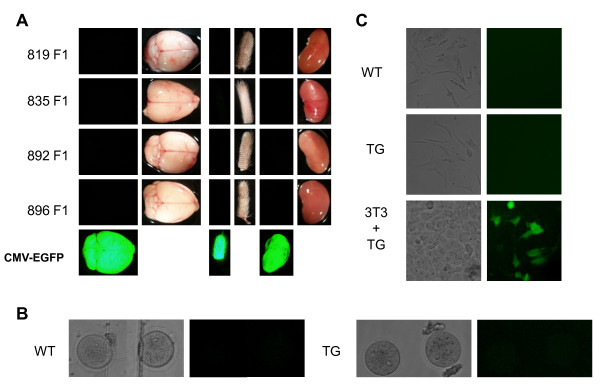
**Characterization of transgenic animals**. **(A) **EGFP expression in brain, tail and kidney of transgenic animals. Bright-field images are shown to illustrate organ morphology. F_1 _generation mice from all transgenic lines were used for the analysis. EGFP expression in transgenic mice carrying a CMV-EGFP transgene (P.S., unpublished results) is shown for comparison. **(B) **EGFP expression in oocytes isolated form wild-type and transgenic animals. Bright-field images are shown to illustrate oocyte morphology. **(C) **EGFP expression in primary fibroblast isolated from wild-type and transgenic animals. NIH3T3 cells transfected with pZMP plasmid were used as positive controls.

Genotyping of transgenic mice should be facilitated by ubiquitous EGFP expression. However, none of the tails of F_0 _mice exhibited EGFP expression originating from the PGK-driven puromycin-IRES-EGFP reporter cassette in the transgene (Figure 4A). Likewise, none of the tested tissues in F_1 _mice (brain, kidney, liver, spleen, testis, and oocytes) showed EGFP expression under the stereomicroscope (Figure 4A and 4B).

To test whether the reporter is completely silenced or the EGFP expression is below a detection limit of our microscope, we isolated tail fibroblasts from transgenic mice and their wild-type siblings and tested in culture their sensitivity to puromycin and assessed the transgene expression by RT-PCR and EGFP fluorescence by flow cytometry and fluorescent microscopy. Results of these experiments confirmed that the reporter cassette in the transgene is silenced in fibroblasts of F_1 _mice of all available transgenic lines (Figure 4C). We also tried to change the genetic background by crossing the C57Bl/6 transgenic animals with BALB-C mice but it did not help to reactivate the silenced reporter in somatic cells (data not shown). This effect is likely due to the epigenetic silencing of the transgene because PCR analysis of genomic DNA showed that the transgene is intact. In addition, transfection of the purified transgene into 3T3 fibroblasts resulted in EGFP expression (Figure 4C) and puromycin resistance (data not shown), further supporting the idea that the transgene is epigenetically silenced.

**Figure 5:**
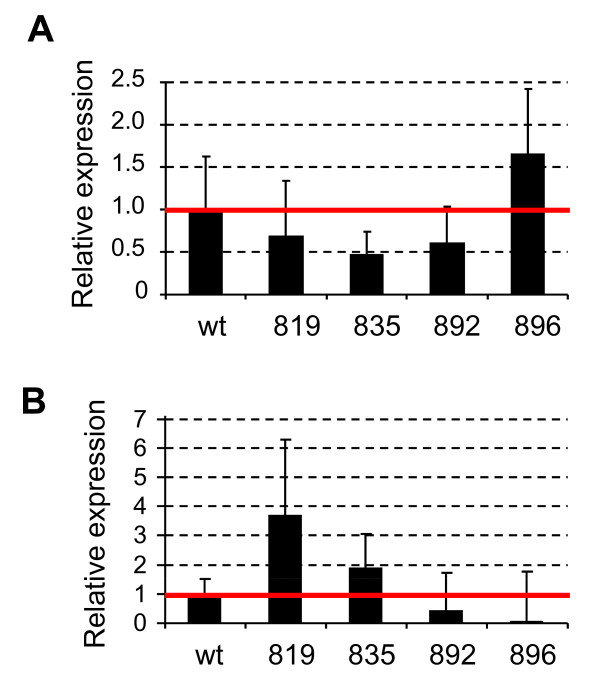
***Single-cell qPCR analysis of *Mos *knock-down and shRNA expression in mouse oocytes***. **(A) **Relative *Mos *mRNA expression in oocytes from transgenic animals (*Mos *mRNA level in wild-type oocytes is set to 1). Rabbit *β-globin *mRNA, which was added to the lysis buffer at the time of collection, was used as an external standard for data normalization. Statistical significance of relative expression changes of *Mos *mRNA levels normalized to the β-globin was analyzed by the pair-wise fixed reallocation randomization test using the REST 2008 software. **(B) **Relative Mos_F shRNA expression in oocytes from transgenic animals. All data are expressed as mean +/- SEM from at least five oocytes.

Although silencing of the reporter cassette in the transgene was disappointing, we analyzed fertility, frequency of parthenogenetic activation, and *Mos *mRNA levels in four available transgenic lines because the shRNA was driven by a different promoter than the puromycin-EGFP reporter and the germline undergoes cycles of epigenetic reprogramming, providing a chance that the transgene would be active in the oocyte. However, oocytes of transgenic animals did not exhibit parthenogenetic activation. Single-cell quantitative real-time PCR (qPCR) showed a possible down-regulation of *Mos *mRNA (up to 2-fold) in transgenic lines #819, #835, and #892 compared to wild-type controls (Figure 5A), but it was not statistically significant when considering the variability of mRNA level in individual oocytes. However, it is possible that a mild down-regulation was induced in the line #835 where we observed the lowest *Mos *mRNA level and qPCR analysis suggested a low level of shRNA expression (Figure 5B). These data indicate that epigenetic silencing affects the whole transgene, leading to low shRNA expression, which in turn is unable to target *Mos *mRNA efficiently.

## Discussion

Long hairpin RNA expression has been a preferred solution for specific gene inhibition by RNAi during oocyte growth and oocyte-to-zygote transition. At least ten different genes were targeted by this approach and strong mRNA knockdown was observed in all cases ([7] and P.S., unpublished results). Successful knockdown in the oocytes with transgenic short hairpin systems was reported in the mouse using Cre-recombination-activated pol III promoter-driven shRNA [31]. A ZP3 promoter-driven shRNA expression in Steppe Lemming oocytes induced an efficient RNAi [32], suggesting that miRNA-like shRNA biogenesis is intact in rodent oocytes.

Here, we show that experiments with pol II-driven miRNA-like shRNA system did not meet expectations and raised questions whether such a system represents a more versatile and economical alternative to the long hairpin RNA-based approach. The expected benefit of the shRNA system, a simple production of the targeting vector, turned out to be correct and targeting vectors were easily produced in a single cloning step. Easy production of different targeting vectors facilitates testing different siRNA sequences in transient cell culture transfections before producing transgenic animals. This used to be an advantage over the long hairpin RNA system, where targeting efficiency of transgenic constructs could be tested only by microinjecting them into incompetent oocytes. To circumvent this problem, different strategies are available now that simplify cloning of long inverted repeats [33] and, in our experience, the transgenic approach with long hairpin RNA is reliable enough that, upon verifying the correct structure of the transgene by sequencing, we routinely proceed directly to production of transgenic animals.

Thus, designing and cloning functional shRNAs is not a significant advantage over producing the traditional long hairpin-expressing transgene. A shRNA with a defined sequence exhibits sequence-specific off-target effects. Thus, one needs at least two different shRNAs and/or other means to assure that off-targeting will not interfere with interpretation of data [34,35]. This complicates the production of transgenic lines because, ideally, one would need to have different transgenic lines expressing different shRNAs targeting the same gene. In addition, obtaining effective shRNAs may also represent a problem. While testing eight different shRNAs designed by the best available algorithms [25-28], we found just two good shRNAs with ~50% knockdown effects in a transient reporter assay. This issue will be reduced in the future as more verified shRNA sequences will become available. Still, obtaining verified shRNAs against oocyte-specific genes might represent a problem.

Transgenic RNAi with shRNA is not more economical. Testing different shRNAs (eight in our case) required custom synthesis of eight long oligonucleotides and cloning of eight targeting vectors plus cloning one targeted reporter vector because the targeted gene was oocyte-specific, hence not expressed in common cell lines. This actually made the total cost of the experiment higher when compared to long hairpin transgenes.

In any case, theoretical advantages became irrelevant during the disappointing pilot experiment, where all transgenic lines produced by traditional pronuclear microinjection carried completely silenced transgenes in all tissues in the F_0 _generation already. In contrast, long hairpin RNAi transgenes produced by pronuclear microinjection in the same transgenic facility, in the same genetic background, and carrying the same ZP3 promoter induced strong knock-down effects in oocytes (PS, unpublished results).

Available evidence points towards the reason for silencing being associated with the shRNA transgene sequence/structure. First, we have never seen such a rapid and complete silencing of a transgene in all tissues of F_0 _animals and their progeny with other transgenes. This silencing is really striking considering the same transgene produces puromycin resistance and EGFP expression when transiently transfected in mouse NIH3T3 cells (Figure 4C). We speculate that, while it is tolerated in cells during transient transfection, the unusual structure of the transgene (flanking with short inverted repeats of LoxP sites and the absence of introns) and expression of unspliced bicistronic reporter mRNAs carrying a viral IRES contribute to its silencing when the transgene is integrated in the genome of an animal. Thus, our data show that optimization of shRNA-expressing transgenes design is needed and that intron-less transgenic cassette compatible with retroviral transgenesis might be suboptimal for transgenic RNAi in the mouse.

## Conclusions

The oocyte-specific transgenic RNAi mediated by shRNA does not have any significant advantage in terms of labour, price, knockdown efficiency, and specificity. Transgenic RNAi with shRNA in the oocyte might represent an advantage only in the case when the same gene is being studied in the oocyte and somatic cells. In other cases, transgenic RNAi with long hairpin RNA appears to be a better approach. Current strategies for cloning long inverted repeats make the production of long hairpin-expressing transgenes feasible and cost-effective [33,36]. To our knowledge, all transgenic RNAi experiments with long hairpin-expressing transgenes yielded transgenic lines with strong silencing including phenocopying the knockout phenotypes. Moreover, detailed analysis of non-specific effects revealed remarkable specificity of transgenic RNAi induced by long hairpin RNA [24], presumably because off-target effects are minimized by processing long dsRNA into a pool of siRNAs with different sequences [37].

## Methods

### Plasmids

#### Renilla-Mos reporters

Renilla-Mos reporters were generated from phRL_SV40 (Promega) by inserting a *Mos *fragment into the *Renilla *3'-UTR. The *Mos *fragment was amplified by PCR from genomic DNA using Mos_XbaI_Fwd and Mos_XbaI_Rev primers (see additional file 1: A list of oligonucleotide sequences used in this study). PCR product was cleaved and inserted into the XbaI site in phRL_SV40 to produce phRL_SV40_mMos and phRL_SV40_ asMos reporters where the *Mos *fragment was inserted in a sense and an antisense orientation, respectively.

#### pLMP and pTMP shRNA plasmids

For each shRNA to be inserted into pLMP and pTMP plasmid, one long oligonucleotide was synthesized (Sigma-Aldrich). Each oligonucleotide was used as a template for PCR (performed according to the manufacturer's instructions) using LMP_oligo.fwd and LMP_oligo.rev primers. Resulting PCR product was digested by EcoRI and XhoI and cloned into the target plasmid digested by XhoI and EcoRI. All plasmids were verified by sequencing.

#### pZMP and pZMP-Mos_F

The pZMP vector was derived from pTMP and pLMP plasmids as follows. 5' and 3' LoxP and NotI sites flanking the transgenic cassette were sequentially inserted into BglII and SalI sites, respectively, in pLMP and pTMP in a form of *in vitro *synthesized annealed linkers (5'loxP.fwd/rev and 3'loxP.fwd/rev, respectively) producing pLMP_LoxP and pTMP_LoxP. Subsequently, the EcoRI site for shRNA cloning in the pLMP_LoxP vector was mutagenized to the MunI site using Quick Change II XL Site-Direct Mutagenesis Kit (Stratagene) according to manufacturer's instructions using LMP_MunI.fwd and LMP_MunI.rev primers.

The ZP3 promoter was amplified from the original transgenic RNAi vector [5] by PCR using primers ZP3_BglII_Fwd and ZP3_BglII_Rev using a Pfu DNA polymerase. The ZP3 promoter-carrying PCR fragment was cleaved by BglII and inserted in the BglII site in the pLMP_LoxP plasmid to get pLMP_LoxP_ZP3 plasmid. The correct orientation of the ZP3 promoter and the absence of mutations were verified by sequencing. Finally, the NotI-flanked CMV-TRE-Puromycin-EGFP cassette in the pTMP_LoxP plasmid was replaced by the NotI site-flanked ZP3 cassette from pLMP_LoxP_ZP3 plasmid to produce the pZMP vector ready for shRNA insertion. The reason for this strategy was that the pTMP_LoxP plasmid did not contain suitable restriction sites for direct insertion of the ZP3 promoter while the pLMP is not a self-inactivating (SIN) retroviral vector and strong promoter present in the 5'LTR region of pLMP would have undesirable effects on shRNA expression.

Finally, Mos_F shRNA was inserted in the pZMP to produce pZMP-Mos_F plasmid. The transgenic cassette (~ 4.5 kb) was released by NotI digest, isolated by Gel Extraction Kit (Qiagen), and purified twice using DNA Clean & Concentrator kit (Zymo Research). The cassette purity and integrity was verified by agarose gel electrophoresis before it was submitted to the transgenic facility.

#### Other plasmids

Cloning of shRNAs targeting firefly luciferase (pGL2, Promega) into pLMP, pTMP and pTRIPZ plasmids (Open Biosystems) was performed as described above using FL_1 primer as a template. The insert for cloning into pSuper vector (OligoEngine) was prepared by annealing oligonucleotides FL_2 and FL_3 and cloning them into BglII and HindIII sites of target vector according to the manufacturer's instructions. A hairpin cloned into the UI2-GFP-SIBR vector [14] was prepared by annealing oligonucleotides FL_4 and FL_5 (see additional file 1: A list of oligonucleotide sequences used in this study). Annealed oligonucleotides were cloned into BpiI-cleaved vector. All plasmids were verified by sequencing.

### Cell culture

#### Transformed cell lines

HeLa, HEK293, and NIH3T3 cells were cultured in Dulbecco's Modified Eagle medium (DMEM, Sigma) supplemented with 10% fetal bovine serum (FBS, Gibco), Penicillin 100 U/ml and Streptomycin 100 μg/ml (Gibco). For transfection, cells were seeded in 24-well plates at the initial density 30,000 (HeLa and NIH3T3) or 60,000 (HEK293) cells per well in 0.5 ml of culture medium. 24 hours later, cells were transfected with 500 ng of plasmid DNA per well. TurboFect (Fermentas) was used as the transfection reagent. pBluescript (Stratagene) was used to equalize the total amount of DNA per transfection. A 1 ml aliquot of fresh culture media was added 6 hours post-transfection. Each transfection was performed at least in duplicates. Cells were collected 48 hours post-transfection and used for analysis.

#### Primary tail fibroblasts culture and puromycin selection

Primary fibroblasts were prepared from tail biopsies by collagenase treatment as described previously [38]. Primary tail fibroblasts from transgenic and wild type mice were cultured in DMEM supplemented with 10% FBS and Penicillin/Streptomycin at 37°C and 5% CO_2 _for at least five days. Before experiment, medium was changed and puromycin was added to the final concentration of 2.5 μg/ml. Cell culture was continued for additional 2 days until the control cells from wild-type mice died.

### Dual Luciferase assay

For luciferase assays, cells were typically transfected with 50-250 ng of a firefly luciferase coding plasmid (pGL4-SV40 or pGL2), 1 ng of a *Renilla *luciferase reporter plasmid, 50 ng of a tested hairpin-coding vector, and pBluescript up to the total DNA amount 500 ng per well. In some experiments, different concentrations of a tested plasmid (20 - 450 ng) were used. Control transfection did not include the shRNA-expressing vector. Cells were harvested 48 hours post-transfection and lysed with 150 μl of Passive Lysis Buffer (Promega). Protein amount in lysates was quantified by Protein Assay Dye Reagent (Bio-Rad) according to the manufacturer's protocol. A 10 μl aliquot of each lysate was pipetted into a 96-well plate and luciferase activity was measured using a Dual-Luciferase Reporter Assay System (Promega) according to the manufacturer's instructions. The measurement was performed on the Modulus Microplate luminometer (Turner BioSystems).

### Mice

#### Production of transgenic founders

All animal experiments were approved by the Institutional Animal Use and Care Committees and were in agreement with Czech law and NIH (National Institutes of Health) guidelines. Transgenic mice were produced in the Transgenic core facility of the Institute of Molecular Genetics Academy of Science of the Czech Republic. Briefly, fertilized donor oocytes were obtained from super-ovulated 3-4 weeks old C57Bl/6N females (Charles Rivers Laboratories). Hormonal stimulation was carried out as follows: 5U of Pregnant Mare's Serum Gonadotropine (PMSG/Folligon; Intervet) was injected into peritoneum. Forty-five hours later, 5U of human Choriogonadotropine (HCG, Sigma) was injected into peritoneum and mice were mated with C57Bl/6N males. One day later, one-cell stage embryos were isolated from plugged females. Pronuclear injection (PNI) of transgene DNA into male pronucleus was performed. Embryo transfer was performed either at one-cell stage directly after PNI or at the two-cell stage after an overnight culture depending on the amount of foster mice available on a specific day. Pseudopregnant CD1 females were used as foster mothers. Females were paired with vasectomized CD1 males (for optimal stimulation of the female) a night before the transfer. Embryos were transferred into the oviduct (15-25 embryos per recipient, into one or both oviducts) under sterile conditions in SPF (specified pathogen free) area of animal house. CD1 mice were obtained from an in-house breeding.

#### Genotyping

The tail biopsies were obtained from 3-4 weeks old mice. GFP expression was analyzed by fluorescent stereomicroscope SZX16 (Olympus). Genotyping was performed by PCR and resulting products were analyzed by electrophoresis on 1.5% agarose gels.

### Oocyte isolation and culture

Fully-grown GV-intact oocytes were obtained from eight-week old mice 44 hours after superovulation by intraperitoneal injection of 0.1 ml (5 units) of PMSG (Folligon; Intervet). Oocytes were collected into M2 medium supplemented with 4 μg of isobutylmethylxanthine (IBMX, 200 mM) to prevent resumption of meiosis. Cumulus cells were removed with a thin glass capillary. Isolated oocytes were either immediately analyzed by microscopy or washed twice in PBS and lysed for single-cell qPCR analysis. GV oocytes used for meiotic maturation were washed five-times in M2 medium without IBMX and cultured overnight in CZB medium supplemented with glutamine (5 μl of 3% glutamine per 1 ml CZB)[39].

### Quantitative real-time RT-PCR (qPCR)

mRNA expression in oocytes was analyzed by single-cell qPCR [40]. Briefly, individual oocytes were washed in PBS and placed separately in 5 μl of water. 1 μg of stuffer rRNA (16S + 23S, Roche) and 15 pg of external standard rabbit β-globin mRNA (Sigma) were added to each sample. All samples were snap-frozen and stored at -80°C until further processing. Before qPCR, samples were incubated at 85°C for 5 minutes to lyse oocytes and then were placed on ice. 1 μl of Oligo(dT) primer (50 μM) or random hexanucleotides (Fermentas) and water up to 13 μl were added to all samples. mRNA was reverse transcribed using RevertAid M-MuLV Reverse transcriptase (Fermentas). Reverse transcriptase was omitted in control (-RT) samples. Resulting cDNA was diluted 3:2 with water and a 3 μl aliquot was used as a template for qPCR. qPCR was performed on the iQ5 machine (Bio-Rad) using Maxima SYBR Green qPCR Master Mix (Fermentas). Specific primers for mouse *Mos *and rabbit β-globin mRNAs were used (see additional file 1: A list of oligonucleotide sequences used in this study). qPCR data were analyzed by the iQ5 software (Bio-Rad) and values of crossing points (CPs) were evaluated for each reaction. PCR efficiency was calculated for each individual reaction using the exponential regression model [41] and CPs values were corrected accordingly. Statistical significance of relative expression changes of *Mos *mRNA levels normalized to the external β-globin standard was analyzed by the pair-wise fixed reallocation randomization test using the REST 2008 software [42].

## Competing interests

The authors declare that they have no competing interests.

## Authors' contributions

LS performed all the experiments. RM participated in the design of the study and data analysis. RS participated in the production of transgenic animals. PS designed and coordinated the study. RM and PS wrote the manuscript. All authors read and approved the final manuscript.

## Supplementary Material

Additional file 1 **A list of oligonucleotide sequences used in this study**. A table listing sequences of all oligonucleotides used in this study. Click here for file
