# Supplementary material for: Shortcomings of short hairpin RNA-based transgenic RNA interference in mouse oocytes
Source: J Negat Results Biomed. 2010 Oct 12;9:8. doi: 10.1186/1477-5751-9-8 (PMC2964603; doi:10.1186/1477-5751-9-8)
Supplement: Additional file 1 — A list of oligonucleotide sequences used in this study. A table listing sequences of all oligonucleotides used in this study. [file 1477-5751-9-8-S1.DOC]

Sarnova et al.

### Additional file 1 – A list of oligonucleotide sequences used in this study

Sequences of oligonucleotides (5’ 🡪 3’) used in our study.

| **Oligonucleotides used for cloning** | |
| --- | --- |
| 5´loxP.fwd | GAT CGC GGC CGC ATA ACT TCG TAT AAT GTA TGC TAT ACG AAG TTA TA |
| 5´loxP.rev | GAT CTA TAA CTT CGT ATA GCA TAC ATT ATA CGA AGT TAT GCG GCC GC |
| 3´loxP.fwd | TCG ACA ATA AAA TAA CTT CGT ATA ATG TAT GCT ATA CGA AGT TAT GCG GCC GCG |
| 3´loxP.rev | TCG ACG CGG CCG CAT AAC TTC GTA TAG CAT ACA TTA TAC GAA GTT ATT TTA TTG |
| LMP_MunI.fwd | CCA ACA GAA GGC TCG AGC AAC CAC AAT TGA AGG GGC TAC TTT AGG AGC AAT TAT CTT GTT TAC |
| LMP_MunI.rev | GTA AAC AAG ATA ATT GCT CCT AAA GTA GCC CCT TCA ATT GTG GTT GCT CGA GCC TTC TGT TGG |
| ZP3_BglII_Fwd | GAC AGA TCT CTG GGA GTT CAA GGC CAG |
| ZP3_BglII_Rev | GAG AGA TCT TAA TGA GAG GCT GAC ACC ACT G |
| LMP_oligo.fwd | CAG AAG GCT CGA GAA GGT ATA TTG CTG TTG ACA GTG AGC G |
| LMP_oligo.rev | CTA AAG TAG CCC CTT GAA TTC CGA GGC AGT AGG CA |
| Mos_Xba_fwd | CAC TCT AGA AGG CAG GGA AGC TCT TCC TG |
| Mos_Xba_rev | GTT TCT AGA GGT CAT CTG CCA CAG GGT G |
| Mos_A | TGC TGT TGA CAG TGA GCG AGG AGA TCC TGA AAG GAG AGA TTA GTG AAG CCA CAG ATG TAA TCT CTC CTT TCA GGA TCT CCG TGC CTA CTG CCT CGG A |
| Mos_B | TGC TGT TGA CAG TGA GCG CCC ATA GAC TGG GAA CAG GTA TTA GTG AAG CCA CAG ATG TAA TAC CTG TTC CCA GTC TAT GGA TGC CTA CTG CCT CGG A |
| Mos_C | TGC TGT TGA CAG TGA GCG AAG GGT TTG GCT CGG TGT ATA ATA GTG AAG CCA CAG ATG TAT TAT ACA CCG AGC CAA ACC CTC TGC CTA CTG CCT CGG A |
| Mos_D | TGC TGT TGA CAG TGA GCG CCC GGA GAT CCT GAA AGG AGA GTA GTG AAG CCA CAG ATG TAC TCT CCT TTC AGG ATC TCC GGA TGC CTA CTG CCT CGG A |
| Mos_E | TGC TGT TGA CAG TGA GCG ATC GGT GTA TAA AGC CAC TTA CTA GTG AAG CCA CAG ATG TAG TAA GTG GCT TTA TAC ACC GAG TGC CTA CTG CCT CGG A |
| Mos_F | TGC TGT TGA CAG TGA GCG CGG GTT TGG CTC GGT GTA TAA ATA GTG AAG CCA CAG ATG TAT TTA TAC ACC GAG CCA AAC CCT TGC CTA CTG CCT CGG A |
| Mos_G | TGC TGT TGA CAG TGA GCG AAC GAC AAC ATA GTT CGG GTT GTA GTG AAG CCA CAG ATG TAC AAC CCG AAC TAT GTT GTC GTG TGC CTA CTG CCT CGG A |
| Mos_H | TGC TGT TGA CAG TGA GCG ACC GAA GAC TCC AAC AGC CTA GTA GTG AAG CCA CAG ATG TAC TAG GCT GTT GGA GTC TTC GGG TGC CTA CTG CCT CGG A |
| **Oligonucleotides used as primers for PCR** | |
| genotyping.fwd | TAA GAA CAG TGG TGT CAG CCT C |
| genotyping.rev | CTC CAG ACT GCC TTG GGA AAA GC |
| globin.fwd | GCA GCC ACG GTG GCG AGT AT |
| globin.rev | GTG GGA CAG GAG CTT GAA AT |
| Mos.fwd | GGG AAC AGG TAT GTC TGA TGC A |
| Mos.rev | CAC CGT GGT AAG TGG CTT TAT ACA |
| shRNA.fwd | GTA CTT TAC AGA ATC GTT GCC |
| shRNA.rev | TAA CCT GAA GAA GTA ATC CCA G |
